# Impact of vitamin D on the prognosis after spinal cord injury: A systematic review

**DOI:** 10.3389/fnut.2023.920998

**Published:** 2023-02-14

**Authors:** Lei Wang, Jinlu Gan, Jingnan Wu, Yingchun Zhou, Deqiang Lei

**Affiliations:** Department of Neurosurgery, Union Hospital, Tongji Medical College, Huazhong University of Science and Technology, Wuhan, Hubei, China

**Keywords:** spinal cord injury, vitamin D, deficiency, neuroprotection, progonosis, insufficiency

## Abstract

Vitamin D (VitD) insufficiency is a worldwide health problem and affects billions of people. Spinal cord injury (SCI) patients seem more susceptible to developing suboptimal levels of VitD. However, the literature regarding its impact on the prognosis of SCI is limited. Thus, in this review, we systematically investigated the published studies *via* a combination of keywords associated with SCI and VitD in four medical databases (Medline, Embase, Scopus, and Web of Science). All included studies were analyzed, and selected clinical data on the prevalence of VitD insufficiency (serum 25-hydroxyvitamin D < 30 ng/ml) and deficiency (serum 25-hydroxyvitamin D < 20 ng/ml) were collected for further meta-analysis *via* random effects. Through literature review, a total of 35 studies were eligible and included. The meta-analysis of VitD status (13 studies, 1,962 patients) indicated high prevalence of insufficiency (81.6% [75.7, 87.5]) and deficiency (52.5% [38.1, 66.9]) after SCI. Besides, low levels of VitD were reported to be associated with a higher risk of skeletal diseases, venous thromboembolism, psychoneurological syndromes, and chest illness after injury. Existing literature suggested that supplemental therapy might act as an adjuvant treatment to facilitate post-injury rehabilitation. Non-human experimental studies highlighted the neuroprotective effect of VitD, which was associated with enhancing axonal and neuronal survival, suppressing neuroinflammation, and modulating autophagy. Therefore, the current evidence suggests that the prevalence of VitD insufficiency is high in the SCI population, and low-level VitD may impair functional restoration after SCI. VitD supplemental treatment may have potential benefits to accelerate rehabilitation in mechanistically related processes after SCI. However, due to the limitation of the available evidence, more well-designed randomized controlled trials and mechanism experimental research are still needed to validate its therapeutic effect, elucidate its neuroprotective mechanism, and develop novel treatments.

## 1. Introduction

Vitamin D (VitD) insufficiency and deficiency are described as a pandemic associated with various chronic diseases in all age populations despite the commercialization of VitD supplements and ongoing prophylaxis projects in the general population (1, 2). The national health and nutrition examination survey in the United States indicated that 23–24% and 64% population had a serum total of 25(OH)D less than 20 and 30 ng/mL, respectively (3, 4). In the European Union, Cashman et al. (5) summarized multiple national surveys and indicated that 40.4% of European individuals had serum 25(OH)D concentrations less than 20 ng/ml on average. Spinal cord injury (SCI) is a potentially devastating event featured by severe sensorimotor deficits and autonomic dysfunction with a high burden on both family and society. SCI patients seem to be more susceptible to VitD insufficiency and deficiency due to an unbalanced diet, co-existing diseases, obesity, and lack of sunlight exposure secondary to physical inactivity (6, 7). Besides, several researchers indicated that post-injury autonomic dysfunction usually caused malfunctioning of endocrinological feedback systems, such as VitD-parathyroid hormone axis, and impaired VitD absorption through skin gastrointestinal tract, which directly led to low levels of VitD in SCI individuals (8–10). However, the mechanism underlying post-SCI VitD level alternation is still poorly understood, and literature summarizing its status and impact on the post-injury prognosis is limited.

Despite its classical role in calcium and phosphorus metabolism, VitD is a neurosteroid and exerts a neuroprotective effect in various neurological diseases. In an animal model of Parkinson’s disease, VitD treatment attenuated the injury of dopaminergic neurons *via* suppressing the release of proinflammatory cytokines and upregulating anti-inflammatory signaling (11). Similar protective findings were also observed in the facial nerve injury model, in which the administration of VitD_3_ could facilitate functional restoration by increasing myelination after 12 weeks of treatment (12).

Recent evidence highlighted the unique value of VitD in post-SCI rehabilitation. VitD deficiency in individuals with SCI has been implicated as a primary etiologic, and environmental factor responsible for multiple musculoskeletal issues (e.g., osteoporosis, fracture, chronic pain, etc.) (6, 7). At the same time, low-level VitD is associated with an increased risk of several neuropsychic diseases (e.g., chronic pain, depression, anxiety, PTSD, etc.) (13, 14). New findings also suggest that VitD insufficiency and deficiency were associated with malfunctioning of autonomic nerve system (e.g., a change of endocrinological feedback systems, impaired cardiac autonomic functions, etc.), which might delay the SCI rehabilitation (8, 15). In addition, the pressure injury, an often-occurring complication after SCI, was also reported to be associated with VitD status (16). Notably, Aminmansour et al. (17) applied a combination therapy of progesterone and VitD in a randomized, double-blinded, placebo-controlled study of acute traumatic SCI. They observed that this treatment plan was associated with better functional outcomes. Nevertheless, the published clinical and experimental studies regarding the effect of VitD in SCI are few, and its therapeutic effect remains to be determined.

In this review, we hypothesized that VitD might play an essential role in post-SCI rehabilitation. To validate this hypothesis, we performed a meta-analysis of published studies regarding the prevalence of VitD insufficiency and deficiency in SCI patients, and systematically summarized the clinical and experimental evidence of VitD’s effect in post-SCI rehabilitation.

## 2. Methods

### 2.1. Search strategy, study selection, and eligibility criteria

This study was performed according to the Preferred Reporting Items for Systematic Reviews and Meta-Analyses guidelines (PRISMA, PRISMA 2020 checklist in supplement materials), and approved by the Ethics Committee of the Tongji Medical College, Huazhong University of Science and Technology (18). Details of this systematic review and meta-analysis were registered on PROSPERO (registration number 2021: CRD42021262207), and can be accessed at^1^. It was a secondary analysis of the completed studies, and the written consent was waived, respectively. A combination of keywords on VitD and SCI and Boolean Operators were applied in four medical databases (Medline, Embase, Scopus, and Web of Science) to retrieve English literature in September 2021. Per the formulated literature search (search strategy in supplement materials), we sought studies (published between 1974 and August 31, 2021) on the VitD status and its impact on the prognosis after spinal cord injury. Both clinical and non-human experimental studies were included in this review to investigate the impact of VitD on the prognosis of SCI and its underlying neuroprotective mechanism.

The retrieved studies were verified and quantified before merging *via* Endnote X9 software (Clarivate Analytics), and the duplicates were removed accordingly. The titles, abstracts, and full texts of identified publications were further evaluated to search for eligible studies. The search was supplemented by the reference list of review articles and selected publications. Reviews, book chapters, case reports, small case series (case number less than 10), conference abstracts, editorial notes, and letters were excluded. Other exclusion criteria included repeated research on the same cohort of patients and unavailable full text. Two independent investigators comprehensively reviewed all selected publications to ensure their eligibility for inclusion in the review. Any disagreement regarding the eligibility of the literature was resolved by thoroughly discussing the publication with a third independent investigator.

### 2.2. Inclusion and exclusion criteria, data extraction, and quality evaluation of meta-analysis

Among the eligible articles, the studies that reported VitD status (VitD deficiency and insufficiency) in the SCI population were selected for meta-analysis. The spinal cord injury (SCI) is defined as damage to the spinal cord resulting from trauma (e.g., result of accident, fall, etc.) or from nontraumatic disease (e.g., degeneration of the spinal column, tumor, etc.). Both traumatic and non-traumatic SCI were included in this review.

The exclusion criteria include (1) studies in a specific population (i.e., athletes), (2) research without qualified data, (3) and the majority of the patients already received VitD treatment. We applied the assessment tool for case series studies from the National Institutes of Health (NIH) to rate the quality of eligible studies in the metanalysis (19). There are nine criteria in the score. For each item, we scored one point for “yes” and zero point for “no,” “not available,” “not reported,” and “cannot determine.” The scores are gauged from all nine criteria (0–9) and generated to represent the overall quality of the study. Data were extracted by two reviewers and included: first author; publication year; country; case number; mean or media age; gender ratio; study design; VitD measure parameter; mean or media injury duration; injury type (acute and chronic); injury extent (incomplete and complete motor function impairment); injury level (paraplegia and tetraplegia); serum VitD status (insufficiency and deficiency).

Based on the international standard for neurological classification spinal cord injury (ISNCSCI), the ASIA Impairment Scale (AIS) is applied to evaluate the injury extent. Unless otherwise stated, complete motor function impairment is used when no motor preserved below the neurological level (AIS A and B) with incomplete motor function impairment referring to AIS C and D (20). Unless otherwise stated, tetraplegia is defined as the impairment or loss of sensorimotor function in the cervical levels of the spinal cord, and paraplegia refers to the impairment or loss of sensorimotor function in the thoracic, lumbar or sacral (but not cervical) segments of the spinal cord (20).

Per clinical practice guidelines of Endocrine Society, we used the serum 25-hydroxyvitamin D [25(OH)D] level to evaluate VitD status in SCI patients (21). We defined VitD insufficiency as serum concentration of 25(OH)D less than 30 ng/ml and VitD deficiency as serum concentration of 25(OH)D less than 20 ng/ml in this study (21, 22).

### 2.3. Statistical analysis

A single-arm meta-analysis was performed *via* Stata (version 15.1). The combined prevalence and 95% confidence interval of VitD deficiency and insufficiency were calculated by the random-effects model. Egger’s and Begger’s tests were performed to assess publication bias, and *p* < 0.05 was considered statistically significant. Subgroup analyses (i.e., case number, age, gender, in/out-patient, acute/chronic injury, injury level, injury duration, injury extent, etc.) were carried out to discuss and clarify the source of heterogeneity.

## 3. Results

### 3.1. Literature selection

**Figure 1:**
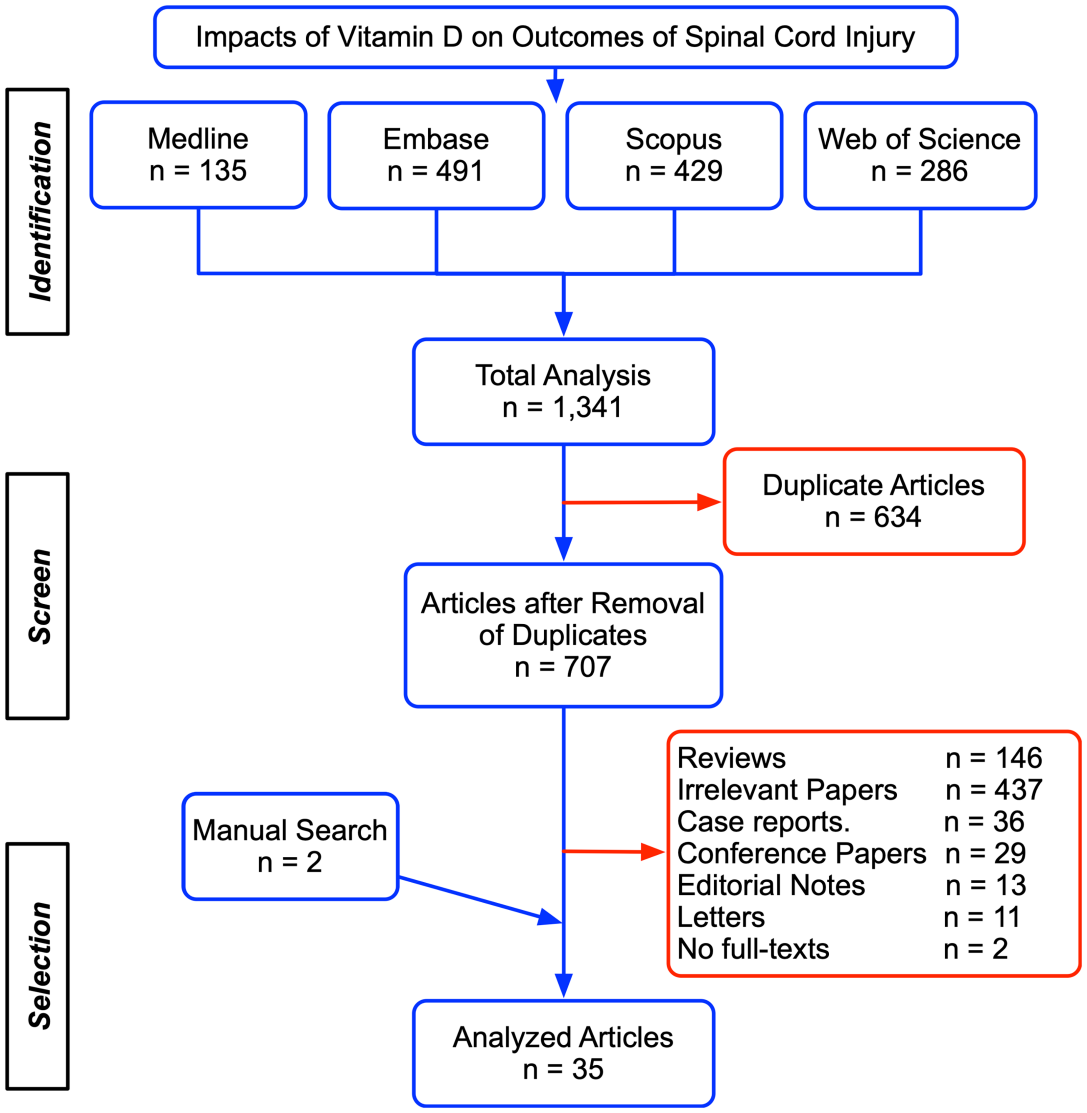
Study selection and characteristics.

We initially identified 1,341 publications after the systematic search in the four databases, including 135 from Medline, 491 from Embase, 429 from Scopus, and 286 from Web of Science. Among these, 634 papers were removed as duplicates and 707 unique studies were assessed for eligibility according to article type, title, abstract, and full texts (Figure 1). Two papers were supplemented from the references list of existing publications. Ultimately, 35 studies were included for the further systematic review and meta-analysis and presented in the supplemental material. There were 21 studies showing post-SCI VitD status (Table 1, (22–34) and Table 2, (35–42)), seven studies regarding the adverse effects associated with a low level of VitD (Table 3, (22, 23, 27, 28, 33, 43, 44)), eight studies that evaluated the potential therapeutic effect of VitD supplement (Table 4 (17, 35, 45–50)), and five non-human experimental researches exploring the underlying mechanism of VitD after SCI (Table 5, (51–55)).

**Table 1:** The summarization of included studies in meta-analysis regarding the prevalence of vitamin D insufficiency and deficiency in SCI patients.

Author, year	Country	Case	Age	Gender (M/F)	Design	Measure.	Participants	Year of injury	Spinal cord injury		Injury extent (Motor)		Injury level		VitD levels	
									Acute	Chronic	Comp.	Incomp.	Tetra.	Para.	Ins.	Def.
Barbonetti, 2016 (23)	Italy	100	51.7	72/28	Cross-section	25(OH)D	Outpatients	6.4	0	100	60	40	39	61	96	78
Bassuino, 2018 (24)	Brazil	39	35.5	39/0	Cross-section	25(OH)D	Outpatients	6.1	0	39	23^a^	16^a^	9	30	34	24
Bauman, 1995 (25)	USA	100	51.0	N.A.	Cross-section	25(OH)D; 1, 25(OH)_2_D	N.A.	20	0	100	N.A.	N.A.	51	49	78	48
Coskun, 2016 (26)	Turkey	42	33.5	28/14	Case–Control	25(OH)D	N.A.	1.4	N.A.	N.A.	10	32	14	28	N.A.	35
Clark, 2020 (27)	USA	253	53.1	208/45	Cohort study	25(OH)D	Outpatients	13.8	0	253	N.A.	N.A.	59	194	195	64
Ehsanian 2019 (28)	USA	282	45.0	202/80	Cohort study	25(OH)D	Inpatients	N.A.	282	0	123	159	144	138	227	N.A.
Garshick, 2019 (22)	USA	312	53.9	260/52	Cross-section	25(OH)D	Outpatients	17.4	0	312	N.A.	N.A.	76	236	237	84
Khammeree 2016 (29)	Thailand	85	N.A.	64/21	Cross-section	25(OH)D	N.A.	N.A.	21	64	34	51	30	55	52	27
Nemunaitis, 2010 (30)	USA	100	48.4	70/30	Case series	25(OH)D	Inpatients	N.A.	100	0	47^a^	53^a^	57	43	93	21^b^
Oleson, 2010 (31)	USA	96	36.8	67/29	Cohort study	25(OH)D	Inpatients & outpatients	N.A.	42^d^	54	96	0	41	55	78^c^	55
Özgirgin, 2016 (32)	Turkey	125	35.2	76/49	Case–control	25(OH)D	Inpatients & outpatients	1.4	45^d^	80	86	39	31	94	119	103
Walia, 2018 (33)	USA	343	54.2	282/61	Cross-section	25(OH)D	Outpatients	17.4	0	343	N.A.	N.A.	82	261	257	93
Waliullah, 2021 (34)	India	85	30.8	60/25	Cross-section	25(OH)D	Inpatients	N.A.	85	0	28^a^	57^a^	0	85	65	50

a It represents the number of patients with complete and incomplete injury.

b This number refers to the patients with severe VitD deficiency (serum 25(OH)D ≤ 10 ng/mL).

c This number refers to the patients with VitD insufficiency (serum 25(OH)D ≤ 32 ng/mL).

d Acute SCI duration < 6 months.

25(OH)D, 25-hydroxyvitamin D; 1, 25(OH)_2_D, 1,25-hydroxyvitamin D; Comp., complete injury; incomp., incomplete; N.A., not available; Ins., insufficiency; Def., deficiency; Tetra, tetraplegia; Para, paraplegia.

**Table 2:** The summary of the literature regarding VitD status after spinal cord injury (not involved in the meta-analysis).

Author, year	Case No.	Design	Population	Main findings
Flueck, 2016 (35)	19	PCS	Elite Athletes with SCI	All participants in the study showed an insufficient or deficient VitD status at the baseline measurement.
Hummel, 2012 (36)	65	CSS	Chronic and traumatic SCI	Disruption of the VitD-PTH axis was prevalent in SCI patients, which might lead to bone loss.
Javidan, 2014 (37)	160	CSS	Chronic and traumatic SCI	In Iranian patients with SCI, there was a high prevalence of VitD deficiency.
Mechanick, 1997 (38)	49	CCS	SCI	The suppressed levels of 1,25(OH)_2−_VitD were more frequently observed in the SCI population (66%).
Pritchett, 2016 (39)	39	CSS	Elite Athletes with SCI	A substantial proportion (~60%) of elite athletes with SCI have insufficient and deficient levels of 25(OH)-VitD in the autumn and winter.
Vaziri, 1994 (40)	40	CCS	Chronic SCI	The serum concentration of calcitriol was significantly lower in the SCI patients. However, there was no significant difference in plasma concentration of 25(OH)-VitD between the SCI and the control group.
Zebracki, 2013 (41)	82	CSS	Pediatric SCI	In comparison with the general pediatric population, pediatric SCI patients exhibited a higher prevalence of VitD insufficiency.
Zhou, 1993 (42)	92	CSS	Chronic SCI	Lower concentration of 25(OH)-VitD was observed in SCI patients, especially in the patients with pressure ulcers.

VitD, vitamin D; SCI, spinal cord injury; CCS, case–control study; CSS, cross-sectional study; PCS, prospective cohort study; PTH, parathyroid hormone.

**Table 3:** Literature summarization of the adverse effects associated with low levels of vitamin D after spinal cord injury.

Author, year	Case No.	Design	Population	Main findings
Barbonetti, 2016 (23)	100	CSS	Chronic SCI	In chronic SCI, low levels of 25(OH)D predicted poor physical function.
Barbonetti, 2017 (43)	100	CSS	Chronic SCI	Among the patients with chronic SCI, serum 25(OH)D levels were inversely associated with depressive symptoms.
Clark, 2020 (27)	253	PCS	Chronic SCI in veterans	In chronic SCI, patients with VitD deficiency had a higher risk of future chest diseases.
Ehsanian, 2019 (28)	282	RCS	Acute inpatient SCI	In acute SCI, without adequate VitD supplement, individuals with low levels of VitD had a higher risk of venous thromboembolism.
Garshick, 2019 (22)	312	CSS	Chronic SCI veterans	No cross-section relationship between VitD deficiency and reduced pulmonary function was observed in this cohort with chronic SCI.
Oleson, 2013 (44)	96	CSS	Acute and chronic SCI	The authors reported a significant correlation between hyperparathyroidism and heterotopic ossification as well as hyperparathyroidism and vitamin D deficiency.
Walia, 2018 (33)	343	CSS	Chronic SCI	The levels of VitD were not associated with respiratory symptoms in chronic SCI.

SCI, spinal cord injury; CSS, cross-sectional study; PCS, prospective cohort study; RCS, retrospective cohort study.

**Table 4:** The summary of clinical studies administrating different vitamin D supplemental regimens in SCI patients.

Author, year	Age (case No)	Gender (M/F)	Design	Injury (years)	Treatment	Therapeutic effect
Aminmansour, 2016 (17)	T: 42 ± 14 (*n* = 32); C: 45 ± 14 (*n* = 32)	T: 19/14; C: 16/16	RCT	Acute SCI within 8 h	Intramuscular injection of progesterone 0.5 mg/kg and oral 25(OH)D_3_ 200 IU/kg twice a day for 5 days on admission	The treatment group had significantly higher motor and sensory function after 6 months of therapy. Early administration (<4 h) showed additional benefits in motor and sensory function recovery.
Bauman, 2005 (45)	Study1: 53 ± 15 (*n* = 10); Study2: 43 ± 13 (*n* = 40)	N.A.	PCS	Study 1: 26 ± 13 years; Study 2: 12 ± 10 years	Study 1: Twice a week 2,000 IU 25(OH)D_3_ for 2 weeks; Study 2: daily 800 IU 25(OH)D_3_ supplementation for 12 months	Serum 25(OH)D_3_ ↑; Serum parathyroid hormone ↓
Bauman, 2005 (46)	T: 43 ± 11 (*n* = 19); C: 42 ± 14 (*n* = 21)	T: 19/0; C: 20/1	RCT	T: 14 ± 10 years C: 9 ± 9 years	4 μg 1α(OH)D_2_ with calcium (1.3 g/d) and 25(OH)D_3_ (800 IU/d) supplementation	Bone mineral density with reduced bone resorption was observed in the lower limb of the treatment group.
Beal, 2018 (47)	47 ± 10 (*n* = 20)	20/0	CCS	17 ± 12 years	Oral intake of 25(OH)D_3_ (213 ± 166 IU, [66–573])	A significant decrease in total cholesterol and improvement in glucose homeostasis were observed in the patients with a high dietary intake of vitamin D.
Chen, 2001 (48)	34 (16–78, *n* = 21)	17/4	RCS	Acute and subacute SCI with bone hyper-resorption: 26 days (6–122)	0.5 μg oral calcitriol once daily throughout the treatment with intravenous administration of 30 mg pamidronate on days 4 through 6 (total of 3 doses)	Serum 1, 25(OH)D_3_ ↑; Serum parathyroid hormone ↑; Bone resorption↓
Flueck, 2016 (35)	37 ± 12 (*n* = 19)	19/0	PCS	Chronic SCI Athletes	6,000 IU daily cholecalciferol supplement over 12 weeks	Serum 25(OH)D_3_ ↑. The treatment improved upper body performance and muscle strength.
Mailhot, 2018 (49)	44.2 ± 16.1 (*n* = 29)	21/8	PCS	Acute and subacute SCI: 29 days (15–90)	1,000 IU daily vitamin D_3_ with weekly additional administration of 10,000 IU vitamin D_3_ in the patients with Vit D insufficiency	The treatment increased serum 25(OH)D_3_ but was unsuccessful in improving the impaired VitD status during inpatient rehabilitation of individuals with a recent SCI.
Pritchett, 2019 (50)	33 ± 15 (*n* = 35)	30/5	PCS	Chronic SCI Athletes:	Patients with sufficient 25(OH)D: 15,000 IU/week of vitamin D_3_ for 12–16 weeks. Patients with insufficiency status: 35,000 IU/week of vitamin D_3_ for the first 4 weeks and 15,000 IU/week for the rest of the study. Patients with deficient status: 50,000 IU/week of vitamin D_3_ for the first 8 weeks and 15,000 IU/week for the rest of the study.	The treatment increased serum 25(OH)D_3_ and improved handgrip strength post supplementation.

T, treatment; C, control; RCT, Randomized Controlled Trial; PCS, prospective cohort study; CCS, case–control study; RCS, retrospective case series; 25(OH)D, 25-hydroxyvitamin D; 1, 25(OH)2D, 1,25-hydroxyvitamin D.

**Table 5:** The literature investigating the neuroprotective mechanism of Vitamin D in non-human experimental spinal cord injury.

Author, year	Species and Cells	Injury	Treatment	Duration	Effect	Mechanism
Bianco, 2011 (51)	Female SD Rats	Compression SCI at T10	Oral delivery of 50 or 200 IU/kg vitamin D_3_	Daily dose for 12 weeks after injury	Ventilatory response to fatigue↑; Normalization of Hoffman reflex	Axon survival within lesion epicenter and distal region
Gueye, 2015 (52)	Male SD Rats	Hemisection SCI at C2	Oral delivery of 500 IU/kg vitamin D_3_	Weekly dose form day 1 or 7 after injury (total for 12 weeks)	Locomotor Function↑; Ventilatory response to fatigue↑; Phrenic nerve response↑	Axon survival in the proximal stump
Gurer, 2017 (53)	Rabbits	I/R injury of spinal cord	Intraperitoneal injections of 0.5 μg/kg calcitriol	Administration for 7 days before SCI	Improvement of histopathological change; Demyelination↓ Neurological function↑;	Caspase-3, Apoptosis↓; Serum and tissue MPO, Inflammation↓; MDA, lipid peroxidation↓; CAT↑ and XO↓, ROS↓
Khajoueinejad, 2019 (54)	Female SD Rats	Contusion SCI at T9-10	Intraperitoneal injections of 1 μg/kg calcitriol	Administration for 7 days after SCI	Improvement of histopathological change; Immunomodulatory effects Neurological function↑;	Motoneurons survival↑; IFN-γ and IL-17A ↓; Leukocytes infiltration↓
Zhou, 2016 (55)	Female SD Rats	Crushing SCI at T9	Intraperitoneal injections of 2 μg/kg calcitriol	Administration for 7 days after SCI	Improvement of histopathological change; Neurological function↑	Motoneurons survival↑; MDA↓, GSH and SOD↑, Oxidative stress↓; Caspase-3, Apoptosis↓; LC3-II and Beclin1↑, Autophagy↑;

SD rats, Sprague–Dawley rats; SCI, spinal cord injury; I/R, ischemia/reperfusion; MPO, myeloperoxidase; MDA, malondialdehyde; CAT, catalase; XO, xanthine oxidase; ROS, reactive oxygen species; GSH, glutathione.

### 3.2. Meta-analysis of vitamin D status in the patients with SCI

#### 3.2.1. Included literature for the meta-analysis

In the 21 studies regarding VitD status after SCI, three studies investigated specific populations (e.g., children, athletes, etc.), one study involved patients receiving extra VitD supplements, and four studies were unqualified for meta-analysis, which were excluded accordingly. Thus, the remaining 13 investigations were included for further analysis, and their details were summarized in Table 1.

#### 3.2.2. The prevalence of insufficient and deficient VitD status after SCI

Among the selected articles, 12 studies reported the incidence of VitD insufficiency, and 11 studies recorded the prevalence of VitD deficiency, which were analyzed, respectively. The quality score of the selected studies varied from 3 to 7 points (Supplementary Table S1).

**Figure 2:**
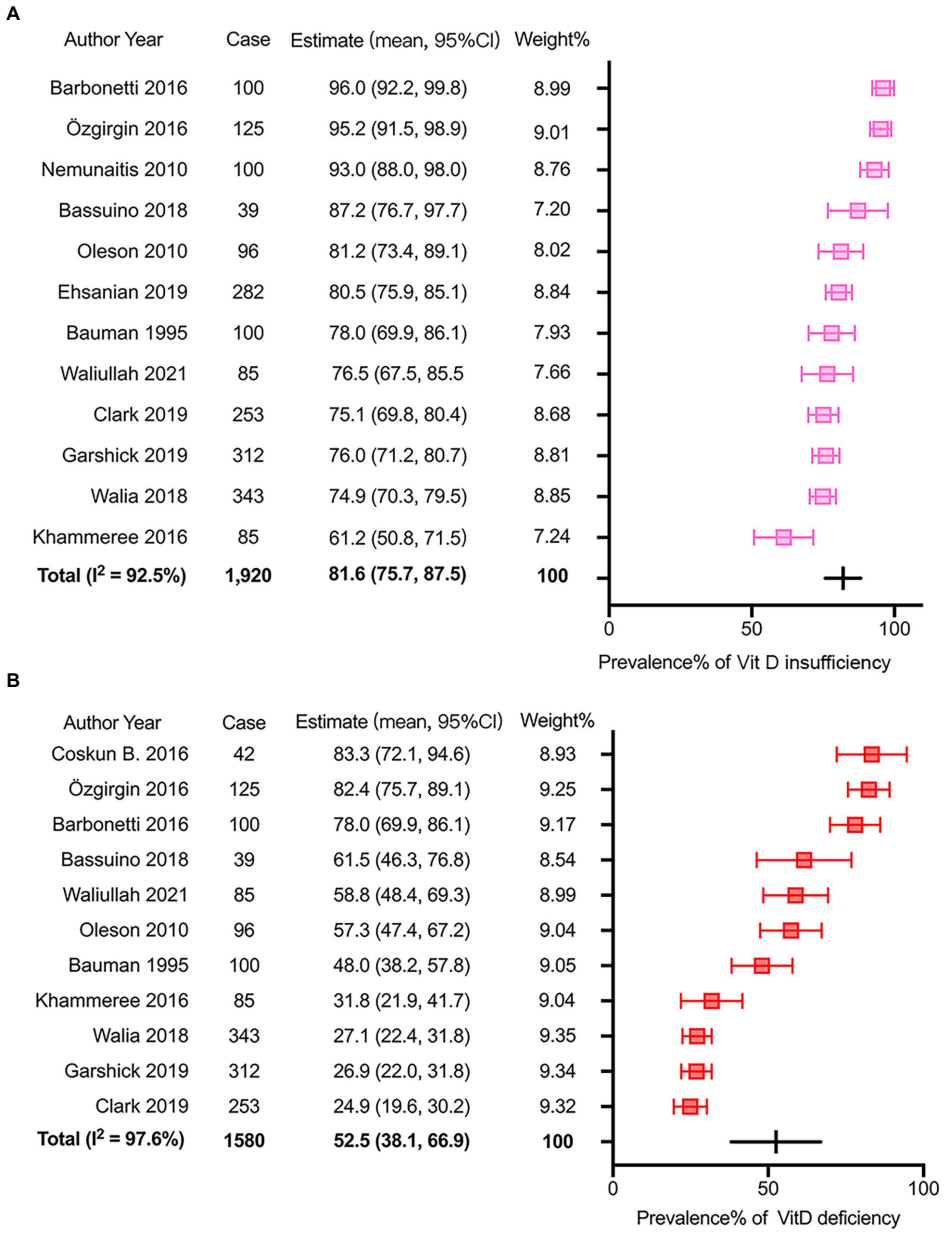
Meta-analysis of the prevalence of insufficient and deficient Vitamin D status after SCI. **(A)** the prevalence of Vitamin D insufficiency after SCI. **(B)** the prevalence of Vitamin D deficiency after SCI.

The prevalence of insufficient VitD status after SCI was reported from 61.2% to 96.0% in the 12 eligible studies with 1,920 participants. The pooled prevalence determined by the random-effects model was 81.6% (95% CI: 75.7–87.5) with significant heterogeneity (*I*^2^ = 92.5%, *p* = 0.00, Figure 2A). The *p* values of Egger’s and Begg’s tests were 0.091 and 0.537 respectively, suggesting no significant publication bias. Subgroup analysis was performed as described (Supplementary Tables S2–S8) to further investigate potential heterogeneity sources. There was more substantial heterogeneity among studies with less than 200 patients, and years of injury less than 10 years. Additionally, insufficient VitD status were more prevalent in the studies with participants less than 200 (patients ≤ 200, *p* = 0.01), more female patients (male/female ≤ 3, *p* < 0.01) and media or mean years of injury less than 10 years (*p* < 0.01).

Regarding the prevalence of VitD deficiency after SCI, 11 articles with 1,580 participants were involved in the meta-analysis. The reported incidence ranged from 24.9 to 83.3%. The overall incidence calculated *via* the random-effects model was 52.5% (95% CI: 38.1–66.9). The I^2^ was 97.6%, which suggested significant heterogeneity among selected studies (*p* < 0.01, Figure 2B). The Egger’s and Begg’s tests exhibited that *p* values were 0.051 and 0.213 respectively, suggesting no significant publication bias. A similar subgroup analysis was performed to evaluate potential heterogeneity (Supplementary Tables S2–S8). More substantial heterogeneity was also observed in the research with less than 200 patients, and years of injury less than 10 years. Besides, VitD deficiency was more severe in the studies with smaller sample size (patients ≤ 200, *p* < 0.01), younger patients (mean or media Age ≤ 46 years, *p* < 0.01), more female patients (male/female ≤ 3, *p* < 0.01), and media or mean years of injury less than 10 years (*p* < 0.01).

### 3.3. Potential adverse impact of VitD insufficiency and deficiency on the post-SCI complications

Through this systematic literature search, we found seven eligible studies regarding on the post-SCI complications which might be associated with low levels of VitD (Table 3). The potential adverse impact included poor physical function (23), depressive symptoms (43), venous thromboembolism (28), heterotopic ossification and hyperparathyroidism (44), chest and respiratory symptoms (22, 27, 33).

### 3.4. Therapeutic effects of vitamin D supplements on the outcome of SCI

Earlier research suggested insufficient levels of VitD were prevalent among persons with SCI. The recommended dietary allowance for VitD is 600 IU/d for ages 1–70 years and 800 IU/d for those >70 years per the dietary reference intake report for calcium and VitD from the Institute of Medicine (56). Supplemental treatments with a dose similar to or higher than the VitD recommended dietary intakes were adopted in SCI patients to maintain an appropriate VitD level. Among the selected studies, there was eight clinical reports exhibiting the therapeutic effects of VitD supplements on the outcome of acute and chronic SCI. The existing literature suggested the VitD supplements had multiple beneficial effects on post-SCI rehabilitation, including correcting dysregulation of VitD-PTH axis (35, 45, 49, 50), reducing bone resorption (46, 48), facilitating motor and sensory functional restoration (17, 35, 50), improving carbohydrate metabolism (47), etc. (Table 4).

### 3.5. Neuroprotective effect of vitamin D in non-human experimental SCI

In the non-human experimental SCI, we retrieved five studies supporting the neuroprotective effect of VitD supplementation (51–55). The proposed mechanisms are multidimensional, involving promoting axonal survival (51, 52), reducing neuronal loss by attenuating oxidative stress (53, 55), suppressing neuroinflammation (54) and modulation of autophagy ((55), Table 5).

## 4. Discussion

In this systematic review, we included a total of 35 studies regarding its impact on the prognosis of SCI. The limited available studies suggest that the prevalence of VitD insufficiency and deficiency is relatively high in the SCI population, which may be associated with delayed neurofunctional restoration and several systematic complications. Besides, the current evidence from clinical and experimental studies shows that VitD supplement treatment may have potential benefits to accelerate rehabilitation in mechanistically related processes after SCI.

### 4.1. The prevalence of insufficient and deficient VitD status after SCI

Previous literature regarding the VitD status after SCI was limited, and existing data remains controversial. Several reports from Veterans Affairs hospitals in the US observed that the prevalence of VitD deficiency was around 25% in chronic SCI populations (22, 27). In contrast, Coskun Benlidayi et al. (26) and Özgirgin et al. (32) reported the rate could reach 80%–90% in SCI participants. We thus performed the meta-analysis in this review to estimate the prevalence of insufficient and deficient VitD status in the general SCI population worldwide. Overall, we retrieved 13 eligible papers with 1,962 participants. Among them, 81.6% of SCI patients had a serum total 25(OH)D less than 30 ng/ml and 52.5% of participants with VitD deficiency. Compared with the general population, pediatric SCI patients exhibited a higher prevalence of VitD insufficiency (41). Additionally, Pritchett et al. (39) noticed that a substantial proportion (~60%) of elite athletes with SCI also had insufficient and deficient levels of 25(OH)D in the autumn and winter. Taken together, there was a high prevalence of VitD insufficiency and deficiency in the SCI population.

### 4.2. Potential adverse impact of VitD insufficiency and deficiency on the post-SCI complications

VitD plays a fundamental role in calcium and phosphate metabolism. Its insufficiency or deficiency is associated with a higher risk of bone diseases (57). Histological investigations recently indicated that VDRs have a wide distribution in non-skeletal tissues, including vessels, skin, muscles, endocrine glands, kidneys, neural tissue, etc., which highlights its unique role in extra-skeletal disease (58). In the SCI population, the previous analysis showed a high prevalence of VitD insufficiency, and deficiency was associated with poor physical functions (23, 59). More importantly, this systematic review showed that VitD abnormality might be associated with several complications, which hampered functional restoration (Table 3).

The low serum VitD is associated with a higher risk of skeletal diseases after SCI (60). In the subjects with long-standing complete SCI, Frotzler et al. (61) observed considerable declines in bone density and a higher risk of historical fractures. Their findings demonstrated that bone loss after SCI could persist for an extended period. The mechanism underlying post-SCI skeletal abnormality is complicated, and recent evidence suggests that the disruption of the VitD-parathyroid hormone (PTH) axis contributes to this pathological process (36, 60). First of all, *via* interaction with VDR, lack of VitD may affect these osteoblasts and osteoclasts and lead to the disruption of bone microstructure and mass. Besides, low-level VitD results in dysregulation of calcium and phosphate homeostasis and abnormal fluctuations of PTH, which has an adverse effect on bones. In the chronic stage of SCI (more than 1 year), Bauman et al. (25) demonstrated a depressed level of VitD and reduction of the serum calcium concentration, which might lead to mild secondary hyperparathyroidism and accelerate the development of osteoporosis. Furthermore, in a study involving 96 SCI individuals, Oleson et al. (44) found that there was a correlation between hyperparathyroidism and heterotopic ossification as well as hyperparathyroidism and VitD deficiency, in which they inferred that low VitD and elevated PTH might increase the risk of heterotopic ossification.

VitD insufficiency and deficiency were also associated with a high risk of venous thromboembolism. Experimental data exhibited that VitD and VDR modulate the expression and activity of multiple coagulation-related proteins (e.g., plasminogen activator inhibitor-1, thrombospondin-1, etc.), which serve as adjunctive antithrombotic agents (62, 63). In a retrospective cohort study involving 282 acute SCI patients, a higher incidence of venous thromboembolism was noted in the subjects with VitD levels <30 ng/ml and an absence of VitD supplementation, which was consistent with the findings in other neurological injuries (28, 64).

Another emerging field of interest regarding VitD-related complications is secondary neurological disorders. VDR is known to express in both neuronal and glial cells in CNS, and VitD is involved in the regulation of neural differentiation and development, modulation of neuroinflammation, maintaining neuroplasticity, and expression of neurotrophins in various physiological and pathological contexts, which provide a rationale for the link between VitD and neurological comorbidities after SCI (65). Barbonetti et al. (43) recently looked into depression after SCI, and they observed that serum 25(OH)D levels were inversely associated with the psychiatric symptoms in the chronic stage. Whereas data is still limited, and future research is needed to clarify the impact of VitD on neurological comorbidity after SCI.

Previous studies in non-SCI populations reported a positive correlation between low VitD levels and increased risk of respiratory diseases (66, 67). Based on this, several research groups looked into the potential association between chest illness and VitD status after SCI. In 2018, Garshick et al. (22) and Walia et al. (33) accessed the cross-sectional associations between respiratory symptoms/pulmonary function and serum VitD levels in chronic SCI patients, and their analysis failed to establish the association. However, the researchers indeed observed that chronic obstructive pulmonary disease and low VitD levels coexisted in some SCI individuals and VitD supplementation might be beneficial in maintaining respiratory health (22, 33). Therefore, to further investigate the association between VitD levels and chest illness, Clark et al. (27) performed a prospective observational study, and they revealed that the reduction of VitD levels might be associated with an increased risk of future chest illness in chronic SCI, particularly in persons with deficient levels. Currently, high-quality evidence is still lacking in the field, and more clinical studies with rigorous design are needed to validate the association between VitD levels and chest illness.

**Figure 3:**
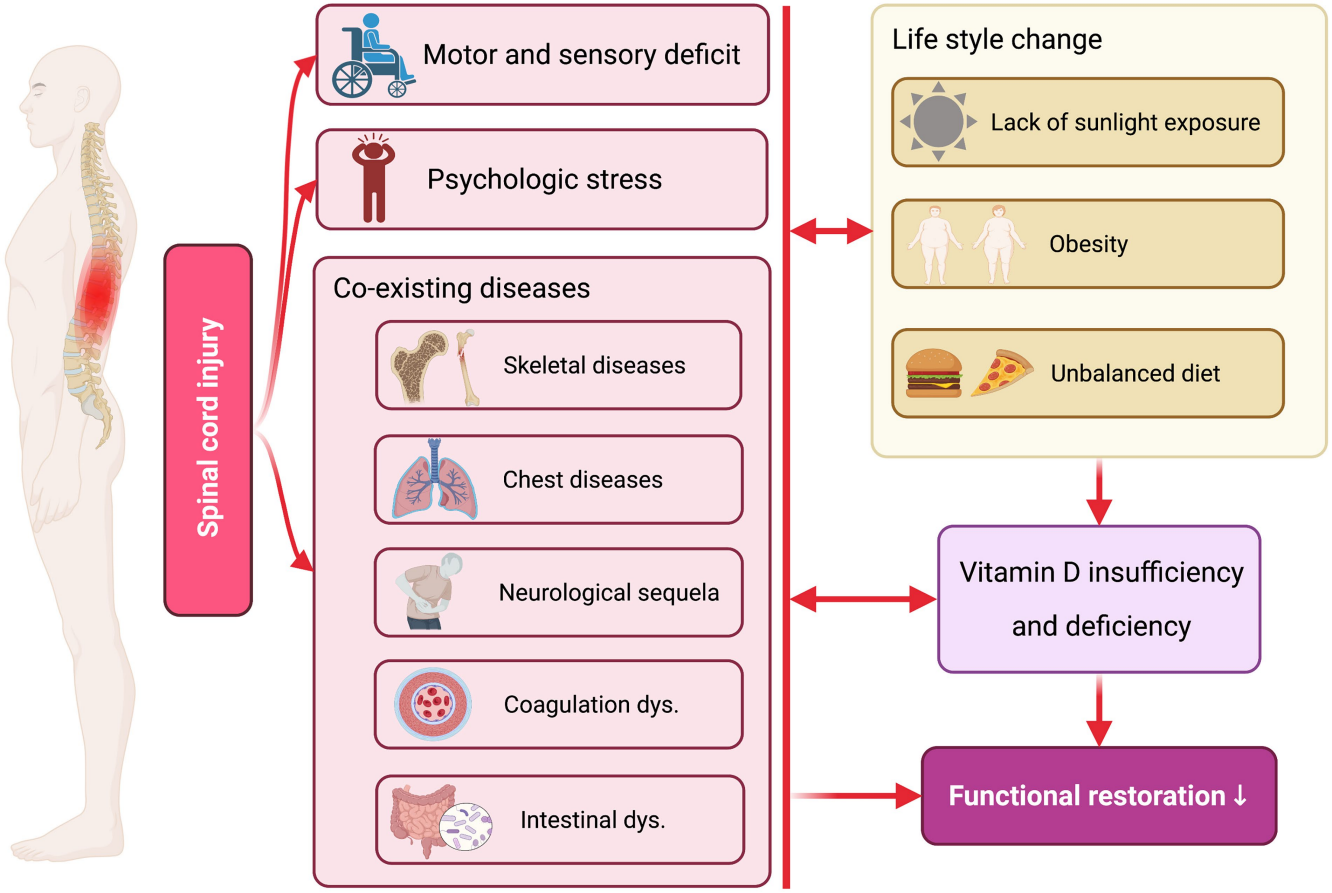
The potential mechanism underlying low Vitamin D levels after SCI. The primary and second injuries after SCI directly lead to severe motor and sensory deficits, psychological stress, and multiple co-existing diseases, which subsequently alter the patients’ lifestyle (e.g., lack of sunlight exposure, obesity, unbalanced diet, etc.). Subsequently, post-SCI functional deficits and unhealthy lifestyles contribute to reduced VitDlevelsl in the patients. Notably, the low levels of VitD can also aggravate functional deficits, psychological stress and co-existing diseases, which start up a vicious cycle and hamper functional restoration after SCI. Created with BioRender.com.

Taken together, we think that SCI patients are at higher risk of developing VitD insufficiency and deficiency, and its underlying mechanism may be associated with the lifestyle change, post-SCI complications, and the corresponding vicious cycle (Figure 3). First of all, the primary and secondary injury in the spinal cord leads to severe motor and sensory dysfunction. Due to the impaired mobility and pain, adequate exposure to sunlight is difficult to achieve in those individuals as most have to stay indoors with decreased physical activity, which has a negative impact on the VitD_3_ synthesis in the skin (68). Secondly, there is converging evidence indicating that dramatic changes in social and family environments after SCI may trigger significant psychiatric stress, which may lead to comorbid psychiatric disorders (such as depression, anxiety, post-traumatic stress disorder, etc.) and makes them more vulnerable to VitD insufficiency (69, 70). It is noteworthy that the researchers also indicated that VitD was a negative acute phase reactant, and post-SCI systematic inflammation might decrease the levels of plasma VDBP, which exacerbated VitD insufficiency (71). Thirdly, SCI patients usually develop multiple complications and comorbidities, such as skeletal diseases, chest illness, neurological sequela, venous thromboembolism, intestinal dysfunction, etc., which may directly result in the lack of VitD or change the lifestyle and affect the level of VitD secondarily (72, 73). For example, persons with SCI are likely to develop pressure ulcers and dysregulation of intestinal microflora with significant diet change, which may affect VitD level synergically *via* impairing its food supply and absorption (42, 74). Notably, the low levels of VitD can also aggravate functional deficits, psychological stress and co-existing diseases, which start up a vicious cycle and hamper functional restoration after SCI.

### 4.3. The therapeutic effects of vitamin D supplements on the outcome of SCI

In the acute and chronic stages of SCI, VitD supplemental treatment can substantially increase VitD concentration and modulate abnormal PTH fluctuation. In 2005, Bauman et al. (45) postulated two supplemental regimens for chronic SCI: the short-term regimen, 2,000 IU 25(OH)D_3_ twice a week for 2 weeks, and the long-term regimen, 800 IU 25(OH)D_3_ daily for 12 months, both of which led to a significant increase of plasma 25(OH)D levels and suppression of plasma PTH. However, they also noticed that the two plans failed to normalize serum VitD levels, which indicated higher doses and longer administration periods were required for the supplementation (45). Notably, later in 2013, they successfully developed an oral regimen for VitD replacement in the chronic SCI population. The patients were administered VitD_3_ at a dose of 2,000 IU daily for 3 months with 1.3 g oral calcium supplementation per day. Normal levels of VitD with a significant decrease in PTH in six of seven participants were restored at the end of the experiment (75). Meanwhile, in recent SCI with complete or incomplete sensorimotor impairments, Mailhot et al. (49) evaluated a VitD repletion protocol, in which participants were given 1,000 IU 25(OH)D_3_ daily for approximately 6 weeks with extra weekly administration of 10,000 IU 25(OH)D_3_ in the patients with VitD insufficiency. They found that the treatment increased serum 25(OH)D_3_ but was unsuccessful in improving the impaired VitD status.

Administration of VitD in SCI facilitates motor and sensory functional restoration. Recently, clinicians performed a randomized trial to assess the effects of progesterone and VitD on functional restoration after acute traumatic SCI. Their findings indicated that the synergic administration improved motor and sensory function after SCI (17). VitD treatment in wheelchair athletes with chronic SCI not only helps them maintain an adequate level of serum VitD but also improves their muscle strength. In a double-blinded study involving 20 indoor wheelchair athletes with VitD insufficiency, researchers administrated 6,000 IU cholecalciferol supplements daily over 12 weeks. The supplemental therapy restored VitD status to an optimal level and seemed to improve upper body performance and muscle strength (35). Similarly, Pritchett et al. (50) adopted a refined and hierarchical protocol pending on the baseline VitD status that exhibited VitD supplementation could increase the serum level of VitD, and improve handgrip strength in the elite athletes with chronic SCI.

There was also evidence supporting the beneficial effect of VitD supplementation on skeletal diseases. The addition of 1α(OH)D_2_ on the basis of routine calcium and VitD supplementation for 1 year was reported to increase the bone mineral density of lower limbs in chronic SCI at 6 months after treatment when compared with the placebo administration (46). In acute and subacute SCI, Chen et al. (48) combined calcitriol and pamidronate therapy, and the treatment significantly inhibited bone hyper-resorption *via* normalization of the VitD-PTH axis. Additionally, a case–control study investigating dietary VitD intakes in chronic SCI suggested that a higher dietary intake of VitD could influence cholesterol and glucose homeostasis, which improved carbohydrate metabolism (47).

### 4.4. Neuroprotective effect of vitamin D in non-human experimental SCI

In the non-human experimental SCI, we retrieved five studies supporting the neuroprotective effect of VitD supplementation (51–55). The proposed mechanisms are multidimensional, involving promoting axonal survival (51, 52), reducing neuronal loss by attenuating oxidative stress (53, 55), suppressing neuroinflammation (54) and modulation of autophagy ((55), Table 5). Oral delivery of VitD for 4 months in a compression SCI model at T10 level improved respiratory adjustment to fatigue and normalized Hoffman reflex *via* increasing the number of axons crossing the lesion site (51). Later, the same research group replicated the findings in a hemisection SCI model at a higher level (C2) (52). Notably, the short-term administration of calcitriol immediately after SCI was also reported to attenuate the histological damage and neuron loss by reducing oxidative stress, inhibiting apoptosis, and promoting autophagy. In addition, pretreatment of calcitriol before SCI also exhibited a protective effect on the ischemia/reperfusion injury of the spinal cord, which was mediated by inhibiting neuronal apoptosis and suppressing regional and general oxidative stress (53). The immunomodulatory property of VitD raises the potential that it may alter the functional status of microglia/macrophages and astrocytes, the key players in the post-SCI neuroinflammation, to improve repairment. Indeed, Khajoueinejad et al. (54) observed that VitD had an immunomodulatory effect on the proliferative response of lymphocytes in the spleen and lymph nodes, which was associated with reduced secretion of proinflammatory cytokines (IFN-γ and IL-17A) and less leukocyte infiltration into the lesion center. However, our knowledge regarding the neuroprotective mechanism of VitD is still limited, and well-designed mechanistic research is needed to elucidate its underlying mechanism.

**Figure 4:**
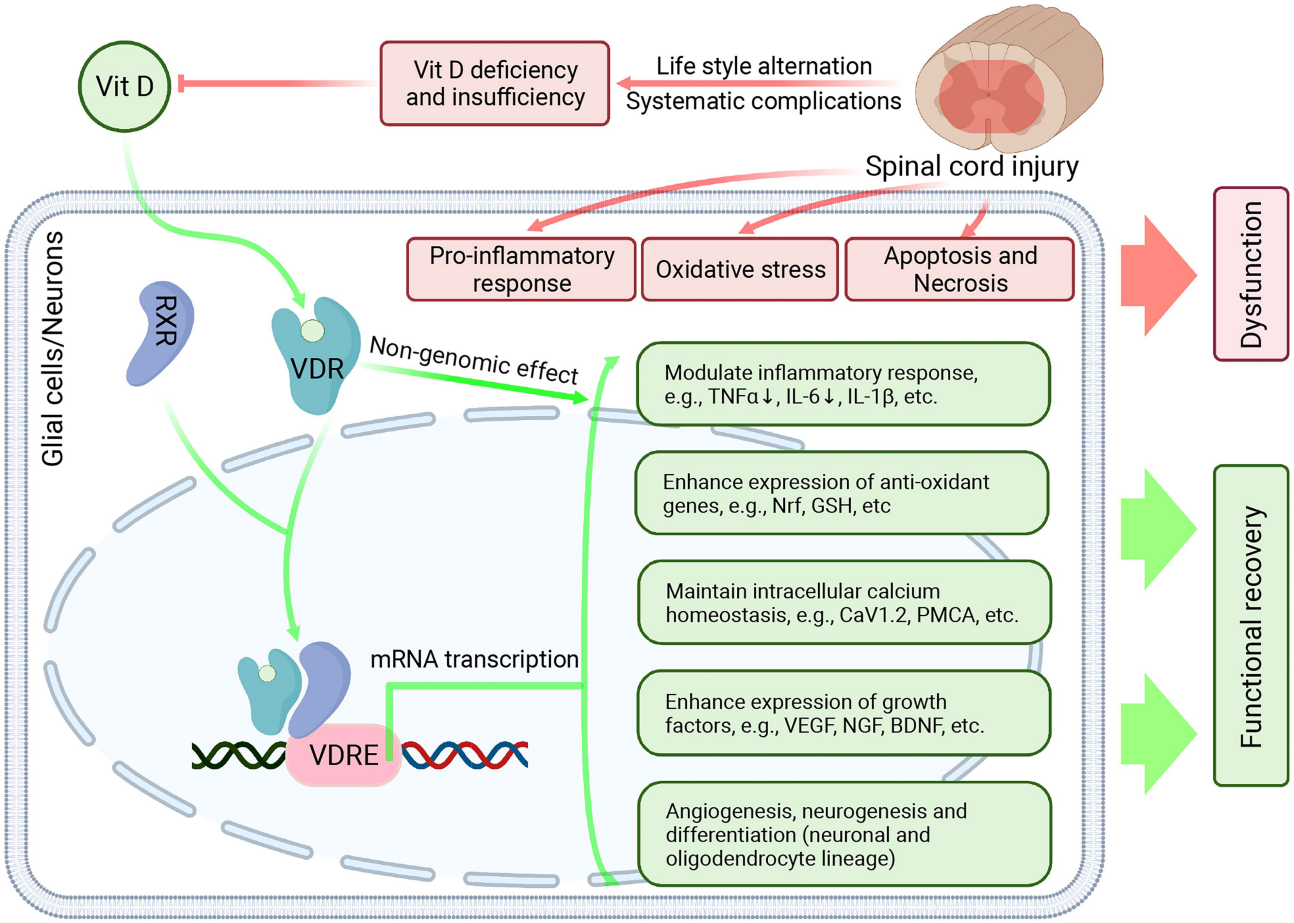
The potential neuroprotective effect of Vitamin D in SCI. After SCI, the primary impact and secondary injury lead to persistent inflammatory response, enhanced oxidative stress, neuronal apoptosis, and necrosis, which results in permanent functional deficits and delays neurofunctional restoration. Besides, due to lifestyle change and systematic complications, the VitD levels were significantly reduced in the SCI individuals. VitD treatment can combat secondary injury after SCI and exert a neuroprotective effect. In the glial cells and neurons, VitD treatment can facilitate its diffusion across the cell membrane. Then VitD binds to VDR and dimerizes with RXR. The VitD-VDR-RXR complex then translocates into the nucleus, binds to VDRE, and promotes the transcription of target genes, which can modulate inflammatory response, attenuate oxidative stress, maintain intracellular calcium homeostasis, enhance the expression of multiple growth factors and neurotrophins, promote angiogenesis and neurogenesis. Created with BioRender.com.

From the evidence of VitD’s effect in both SCI and other neurological disease models, we speculated that the potential neuroprotective effect of Vitamin D might be mediated by its genomic and non-genomic effects (Figure 4). First of all, VitD diffuses through the cell membrane, binds to VDR, and dimerizes with RXR, which then translocates into the nucleus and binds to VitD response element (VDRE). VDRE locates on a large number of genes and its binding with VitD-VDR-RXR complex leads to the transcription of target genes, which could modulate inflammatory response (54, 76), attenuate oxidative stress *via* Nrf2 and Klotho pathway (53, 55, 77, 78), maintain intracellular calcium homeostasis (79), enhance the expression of multiple growth factors and neurotrophins (e.g., vascular endothelial growth factor [VEGF], brain-derived neurotrophic factor [BDNF], etc.) (80), promote axonal regeneration, angiogenesis and neurogenesis (51, 52) (81), modulation of autophagy (55), etc. (Figure 4). Besides, it was recognized that VitD could exert an immediate non-genomic effect through membrane VDR in CNS, which then modulated the functional status of calcium-and kinase-activated signaling pathways (82).

### 4.5. Future direction

In this systematic review, we retrieved a multi-level of evidence supporting the beneficial effects of VitD on post-SCI rehabilitation. However, the neuroprotective mechanisms of VitD have not been elucidated by current experimental studies, and most clinical studies in the field are observational and small-scale, which limits its wide application.

In the experimental research, more in-depth *in vitro* and *in vivo* investigations are needed to explicate the precise molecular mechanism underlying the neuroprotective effect of VitD. In the adult brain, VitD has both genomic and non-genomic actions on various neurological functions (82). It remains to be seen whether VitD could exert neuroprotection *via* similar VDR-depended mechanisms in the context of SCI. In particular, VitD modulated inflammatory response in CNS and immune systems (83). Future researchers should elucidate the exact effect of VitD in the systematic inflammation and neuroinflammation after SCI (84). Furthermore, VitD can enhance neural stem cell proliferation and differentiation into neurons and oligodendrocytes (85, 86). It would be interesting to explore whether VitD could interact with neural stem cells to promote neurons and myelin regeneration after SCI *via* enhancing and modulating endogenous neurogenesis (87–89). Recent literature indicated that small molecules combined with collagen hydrogel directed neurogenesis and migration of neural stem cells after implantation in the lesioned spinal cord (90). As consecutive systematic administration of VitD is known to have poor delivery efficiency in the lesion site and lead to adverse effects in a large dose, topical application of VitD treatment *via* a combination of hydrogel, small molecules, cells, and other small-releasing systems is a promising therapeutic strategy towards SCI.

In future clinical studies, clinicians should design large-scale, double-blinded, and random-control trials to validate the therapeutic effect of VitD as an adjuvant treatment on the functional restoration following SCI. We should explore and standardize the administration protocol of VitD monitoring and supplementation (e.g., dose, therapeutic window, duration, etc.) in the acute and chronic stages of SCI. Another interesting direction is combining VitD treatment with other micronutrients and medicine (e.g., progesterone, Vitamin E, Vitamin C, etc.) and determining whether they have a synergic effect on the improvement of SCI rehabilitation (17, 91).

### 4.6. Limitations

Our systematic review with meta-analysis has several limitations. First, in the meta-analysis of the prevalence of VitD insufficiency and deficiency, there is high heterogeneity among included studies. To diminish its impact on the validity of the results, a single-arm meta-analysis with random effect was applied. We also performed the subgroups analysis to explore the source of heterogeneity and identified more substantial heterogeneity among studies with patients less than 200 and years of injury less than 10 years. However, the high heterogeneity inevitably limits the outcomes of this meta-analysis. Second, in the systematic review, most of the included clinical papers are observational, hospital-based, and performed in different countries, which can introduce potential bias. Third, in the clinical studies, the inclusion and exclusion criteria vary, and the participants have different baseline levels of VitD, which may bias the results.

## 5. Conclusion

Based on the discovered protective and bioactive effect of VitD on neurological disorders, we hypothesize that VitD might play an essential role in post-SCI rehabilitation. Through this meta-analysis and systematic review, we retrieved multi-level evidence that supported this hypothesis, including (1) there was a high prevalence of VitD insufficiency and deficiency in the SCI population, (2) low-level of VitD was associated with several complications, which hampered the functional restoration, (3) the supplement treatment might have potential benefits to accelerate rehabilitation in mechanistically related processes after SCI. However, due to the limitation of the evidence, our results should be interpreted carefully, and more well-designed randomized controlled trials and mechanism experimental research are needed to validate its therapeutic effect, elucidate its neuroprotective mechanism, and develop novel treatments.

## Data availability statement

The original contributions presented in the study are included in the article/supplementary material, further inquiries can be directed to the corresponding authors.

## Author contributions

LW and DL: conceptualization, data curation, funding acquisition, project administration, supervision, writing—original draft, and writing—review and editing. LW, JG, JW, and YZ: formal analysis, investigation, methodology, resources, software, validation, and visualization. All authors contributed to the article and approved the submitted version.

## Funding

This work was supported by National Nature Science Foundation of China (grant number 81771334), Natural Science Foundation of Hubei Province of China (grant number 2017CFB706) and Free Innovation Fund of Wuhan Union Hospital (grant number 2021xhyn105). The funders had no role in study design, data collection and analysis, decision to publish, or preparation of the paper.

## Conflict of interest

The authors declare that the research was conducted in the absence of any commercial or financial relationships that could be construed as a potential conflict of interest.

## Publisher’s note

All claims expressed in this article are solely those of the authors and do not necessarily represent those of their affiliated organizations, or those of the publisher, the editors and the reviewers. Any product that may be evaluated in this article, or claim that may be made by its manufacturer, is not guaranteed or endorsed by the publisher.

## Supplementary material

The Supplementary material for this article can be found online at: https://www.frontiersin.org/articles/10.3389/fnut.2023.920998/full#supplementary-material

## Footnotes

^1^ www.crd.york.ac.uk/prospero/display_record.php?RecordID=262207

## Abbreviations

BDNF: Brain-derived neurotrophic factor
CNS: Central nerves system
IL: Interleukin
NIH: National Institutes of Health
NMDA: N-methyl-D-aspartic acid
PTH: Parathyroid hormone
RXR: Retinoid X receptor
SCI: Spinal cord injury
TNF-α: Tumor necrosis factor-alpha
VDBP: Vitamin D binding protein
VDR: Vitamin D receptor
VDRE: Vitamin D response element
VEGF: Vascular endothelial growth factor
VitD: Vitamin D
UVB: Ultraviolet B
