# Supplementary material for: Impact of vitamin D on the prognosis after spinal cord injury: A systematic review
Source: Front Nutr. 2023 Feb 14;10:920998. doi: 10.3389/fnut.2023.920998 (PMC9973556; doi:10.3389/fnut.2023.920998)
Supplement: Supplementary file 1 [file Data_Sheet_1.DOCX]

Title: Impact of Vitamin D on the prognosis after spinal cord injury: a systematic review

- Limitation: English literatures.
- Date: From 1974 to August 31^th^, 2021
- Search strategy:
- Ovid-Medline: 135 references

Vitamin D:

#1: Vitamin D/ OR Vitamin D.mp.

#2: Ergocalciferols/ OR Vitamin D2.mp.

#3: Cholecalciferol/ OR Vitamin D3.mp.

#4: Calcitriol/ OR Calcitriol.mp.

#5: Calcifediol/ OR Calcifediol.mp.

#6: #1 OR #2 OR #3 OR #4 OR #5

Spinal cord injury

#7: Spinal Cord Injuries/ or spinal cord injury.mp.

#8: Spinal Cord Compression/ or spinal cord compression.mp.

#9: Spinal Cord Contrusion.mp.

#10 Spinal Cord Hemisection.mp.

#11: #7 OR #8 OR #9 OR #10

Overall: #6 and #11

- Ovid-Embase: 491 references

#1: Vitamin D/ OR Vitamin D.mp.

#2: Ergocalciferols/ OR Vitamin D2.mp.

#3: Cholecalciferol/ OR Vitamin D3.mp.

#4: Calcitriol/ OR Calcitriol.mp.

#5: Calcifediol/ OR Calcifediol.mp.

#6: #1 OR #2 OR #3 OR #4 OR #5

Spinal cord injury

#7: Spinal Cord Injuries/ or spinal cord injury.mp.

#8: Spinal Cord Compression/ or spinal cord compression.mp.

#9: Spinal Cord Contrusion.mp.

#10: Spinal Cord Hemisection.mp.

#11: #7 OR #8 OR #9 OR #10

Overall: #6 and #11

- Scopus:429 references

TITLE-ABS-KEY ("Vitamin D" OR "Vitamin D2" OR "Vitamin D3" OR cholecalciferol OR calcitriol OR calcifediol OR ergocalciferols)

AND

TITLE-ABS-KEY ((spinal AND cord AND injury) OR (spinal AND cord AND compression) OR (spinal AND cord AND contusion) OR (spinal AND cord AND hemisection))

- Web of science: 286 references

Ts=("Vitamin D" OR "Vitamin D2" OR "Vitamin D3" OR cholecalciferol OR calcitriol OR calcifediol OR ergocalciferols)

AND

- Ts=((spinal AND cord AND injury) OR (spinal AND cord AND compression) OR (spinal AND cord AND contusion) OR ( spinal AND cord AND hemisection))
