# Supplementary material for: Impact of vitamin D on the prognosis after spinal cord injury: A systematic review
Source: Front Nutr. 2023 Feb 14;10:920998. doi: 10.3389/fnut.2023.920998 (PMC9973556; doi:10.3389/fnut.2023.920998)
Supplement: Supplementary file 2 [file Data_Sheet_2.DOCX]

| **Supplementary Table S1 Quality Assessment for Case Series Studies included in the meta-analysis.** | | | | | | | | | | |
| --- | --- | --- | --- | --- | --- | --- | --- | --- | --- | --- |
| **Study** | **Criteria** | | | | | | | | | **Total** |
|  | **Was the study question or objective clearly stated?** | **Was the study population clearly and fully described, including a case definition?** | **Were the cases** **consecutive?** | **Were the subjects comparable?** | **Was the intervention clearly described?** | **Were the outcome measures clearly defined, valid, reliable and implemented consistently across all study participants?** | **Was the length of follow-up adequate?** | **Were the statistical methods well described?** | **Were the results well described?** |  |
| Barbonetti, 2016A(1) | 1 | 1 | 1 | 0 | 0 | 1 | 0 | 1 | 1 | 6 |
| Bassuino, 2018(2) | 1 | 1 | 1 | 0 | 0 | 1 | 0 | 1 | 1 | 6 |
| Bauman, 1995(3) | 1 | 1 | 1 | 0 | 0 | 1 | 0 | 1 | 1 | 6 |
| Coskun Benlidayi, 2016(4) | 1 | 1 | 1 | 1 | 0 | 1 | 0 | 1 | 1 | 7 |
| Clark, 2019(5) | 1 | 1 | 1 | 0 | 0 | 1 | 1 | 1 | 1 | 7 |
| Ehsanian 2019(6) | 1 | 1 | 1 | 0 | 0 | 1 | 0 | 1 | 1 | 6 |
| Garshick, 2019(7) | 1 | 1 | 1 | 0 | 0 | 1 | 0 | 1 | 1 | 6 |
| Khammeree 2016(8) | 1 | 1 | 1 | 0 | 0 | 1 | 0 | 1 | 1 | 6 |
| Nemunaitis, 2010(9) | 1 | 1 | 1 | 0 | 0 | 1 | 0 | 1 | 1 | 6 |
| Oleson, 2010(10) | 1 | 1 | 1 | 0 | 0 | 1 | 0 | 1 | 1 | 6 |
| Özgirgin, 2016(11) | 1 | 1 | 1 | 0 | 0 | 1 | 0 | 1 | 1 | 6 |
| Walia, 2018(12) | 1 | 1 | 1 | 0 | 0 | 1 | 0 | 1 | 1 | 6 |
| Waliullah, 2021(13) | 1 | 1 | 1 | 0 | 0 | 1 | 0 | 1 | 1 | 6 |

1. Barbonetti, A., Sperandio, A., Micillo, A., D'Andrea, S., Pacca, F., Felzani, G., et al. (2016). Independent Association of Vitamin D With Physical Function in People With Chronic Spinal Cord Injury. *Archives of Physical Medicine and Rehabilitation* 97(5)**,** 726-732. doi: 10.1016/j.apmr.2016.01.002.

2. Bassuino, M.S., Kaminski, E.L., Garcia, L.O., Linden, R., Antunes, M.V., Schneider, R.H., et al. (2018). Factors related to decreased vitamin D levels in men with spinal cord injury living in a subtropical region. *Scientia Medica* 28(2). doi: 10.15448/1980-6108.2018.2.28381.

3. Bauman, W.A., Zhong, Y.G., and Schwartz, E. (1995). Vitamin D deficiency in veterans with chronic spinal cord injury. *Metabolism-Clinical and Experimental* 44(12)**,** 1612-1616. doi: 10.1016/0026-0495(95)90083-7.

4. Coskun Benlidayi, I., Basaran, S., Seydaoglu, G., and Guzel, R. (2016). Vitamin D profile of patients with spinal cord injury and post-stroke hemiplegia: All in the same boat. *J Back Musculoskelet Rehabil* 29(2)**,** 205-210. doi: 10.3233/BMR-150615.

5. Clark, K., Goldstein, R.L., Hart, J.E., Teylan, M., Lazzari, A.A., Gagnon, D.R., et al. (2020). Plasma vitamin D, past chest illness, and risk of future chest illness in chronic spinal cord injury (SCI): a longitudinal observational study (vol 10, pg 125, 2020). *Spinal Cord* 58(4)**,** 513-513. doi: 10.1038/s41393-020-0437-3.

6. Ehsanian, R., Timmerman, M.A., Wright, J.M., McKenna, S., Dirlikov, B., and Crew, J. (2019). Venous Thromboembolism is Associated With Lack of Vitamin D Supplementation in Patients With Spinal Cord Injury and Low Vitamin D Levels. *Pm&R* 11(2)**,** 125-134. doi: 10.1016/j.pmrj.2018.09.038.

7. Garshick, E., Walia, P., Goldstein, R.L., Teylan, M.A., Lazzari, A.A., Tun, C.G., et al. (2019). Associations between vitamin D and pulmonary function in chronic spinal cord injury. *Journal of Spinal Cord Medicine* 42(2)**,** 171-177. doi: 10.1080/10790268.2018.1432305.

8. Khammeree, T., Vichiansiri, R., Sawanyawisuth, K., and Manimmanakorn, N. (2016). Vitamin D abnormalities in Thai patients with spinal cord injuries. *Asian Biomedicine* 10(6)**,** 595-601. doi: 10.5372/1905-7415.1006.528.

9. Nemunaitis, G.A., Mejia, M., Nagy, J.A., Johnson, T., Chae, J., and Roach, M.J. (2010). A descriptive study on vitamin D levels in individuals with spinal cord injury in an acute inpatient rehabilitation setting. *Pm r* 2(3)**,** 202-208; quiz 228. doi: 10.1016/j.pmrj.2010.01.010.

10. Oleson, C.V., Patel, P.H., and Wuermser, L.-A. (2010). Influence of Season, Ethnicity, and Chronicity on Vitamin D Deficiency in Traumatic Spinal Cord Injury. *Journal of Spinal Cord Medicine* 33(3)**,** 202-213. doi: 10.1080/10790268.2010.11689697.

11. Özgirgin, N., Koyuncu, E., Nakipoğlu Yüzer, G.F., Taşoğlu, Ö., and Yenigün, D. (2016). Is spinal cord injury a risk factor for vitamin D deficiency? *Turkiye Fiziksel Tip ve Rehabilitasyon Dergisi* 62(1)**,** 57-63. doi: 10.5606/tftrd.2016.39260.

12. Walia, P., Goldstein, R.L., Teylan, M., Lazzari, A.A., Hart, J.E., Tun, C.G., et al. (2018). Associations between vitamin D, adiposity, and respiratory symptoms in chronic spinal cord injury. *Journal of Spinal Cord Medicine* 41(6)**,** 667-675. doi: 10.1080/10790268.2017.1374020.

13. Waliullah, S., Kumar, D., Kumar, D., Tewari, P.G., Kumar, V., and Srivastava, R.N. (2021). Prevalence of Vitamin D Deficiency in a Young Adult With Acute Spinal Cord Injury. *Cureus* 13(3). doi: 10.7759/cureus.13791.
