# Supplementary material for: Impact of vitamin D on the prognosis after spinal cord injury: A systematic review
Source: Front Nutr. 2023 Feb 14;10:920998. doi: 10.3389/fnut.2023.920998 (PMC9973556; doi:10.3389/fnut.2023.920998)
Supplement: Supplementary file 3 [file Data_Sheet_3.DOCX]

Supplement Table S2. Subgroup analysis of case number in meta-analysis

|  | Patients > 200 | | | | Patients ≦ 200 | | | |
| --- | --- | --- | --- | --- | --- | --- | --- | --- |
| Symptoms | No. | Estimate (95% C.I.) | I^2^(%) | P | No. | Estimate (95% C.I.) | I^2^(%) | P |
| VitD ins. | 4 | 76.7 (74.1, 79.3) | 16.7 | 0.31 | 8 | 84.4 (77.4, 91.3) | 90.0 | <0.01 |
| VitD def. | 3 | 26.4 (23.5, 29.3) | 0.00 | 0.80 | 8 | 62.7 (49.6, 75.9) | 93.3 | <0.01 |

Abbreviations: Ins., insufficiency; def. deficiency; C.I., confidence interval.

Supplement Table S3. Subgroup analysis of age in meta-analysis

|  | Mean or media Age>46 years | | | | Mean or media Age ≦ 46 years | | | |
| --- | --- | --- | --- | --- | --- | --- | --- | --- |
| Symptoms | No. | Estimate (95% C.I.) | I^2^(%) | P | No. | Estimate (95% C.I.) | I^2^(%) | P |
| VitD ins. | 6 | 82.3 (73.7, 90.9) | 94.4 | <0.01 | 5 | 84.4 (76.3, 92.5) | 88.0 | <0.01 |
| VitD def. | 5 | 40.7 (24.3, 57.1) | 97.3 | <0.01 | 5 | 69.0 (56.7, 81.4) | 86.5 | <0.01 |

Abbreviations: Ins., insufficiency; def. deficiency; C.I., confidence interval.

Supplement Table S4. Subgroup analysis of gender ratio in meta-analysis

|  | Male/female > 3 | | | | Male/female ≦ 3 | | | |
| --- | --- | --- | --- | --- | --- | --- | --- | --- |
| Symptoms | No. | Estimate (95% C.I.) | I^2^(%) | P | No. | Estimate (95% C.I.) | I^2^(%) | P |
| VitD ins. | 5 | 75.0 (70.0, 80.0) | 67.1 | 0.02 | 6 | 87.7 (81.3, 94.1) | 89.5 | <0.01 |
| VitD def. | 5 | 31.3 (24.5, 38.1) | 80.7 | <0.01 | 5 | 72.2 (61.4, 83.0) | 86.0 | <0.01 |

Abbreviations: Ins., insufficiency; def. deficiency; C.I., confidence interval.

Supplement Table S5. Subgroup analysis of years of injury in meta-analysis

|  | Years of injury > 10 | | | | Years of injury ≦ 10 | | | |
| --- | --- | --- | --- | --- | --- | --- | --- | --- |
| Symptoms | No. | Estimate (95% C.I.) | I^2^(%) | P | No. | Estimate (95% C.I.) | I^2^(%) | P |
| VitD ins. | 4 | 75.6 (73.0, 78.3) | 0.0 | 0.92 | 6 | 88.6 (82.4, 94.9) | 88.2 | <0.01 |
| VitD def. | 4 | 30.5 (23.5, 37.5) | 83.0 | <0.01 | 5 | 73.6 (64.1, 83.2) | 79.1 | <0.01 |

Abbreviations: Ins., insufficiency; def. deficiency; C.I., confidence interval.

Supplement Table S6. Subgroup analysis of injury extent in meta-analysis

|  | Complete/incomplete > 1 | | | | Complete/incomplete ≦ 1 | | | |
| --- | --- | --- | --- | --- | --- | --- | --- | --- |
| Symptoms | No. | Estimate (95% C.I.) | I^2^(%) | P | No. | Estimate (95% C.I.) | I^2^(%) | P |
| VitD ins. | 4 | 91.1 (85.2, 97.0) | 77.3 | <0.01 | 4 | 78.5 (67.1, 89.8) | 91.6 | <0.01 |
| VitD def. | 4 | 70.6 (58.5, 82.8) | 85.4 | <0.01 | 3 | 57.9 (28.8, 87.0) | 95.6 | <0.01 |

Abbreviations: Ins., insufficiency; def. deficiency; C.I., confidence interval.

Supplement Table S7. Subgroup analysis of injury level in meta-analysis

|  | Tetra./para. > 0.5 | | | | Tetra./para. ≦ 0.5 | | | |
| --- | --- | --- | --- | --- | --- | --- | --- | --- |
| Symptoms | No. | Estimate (95% C.I.) | I^2^(%) | P | No. | Estimate (95% C.I.) | I^2^(%) | P |
| VitD ins. | 6 | 82.4 (73.7, 91.0) | 92.3 | <0.01 | 6 | 80.8 (72.3, 89.3) | 93.0 | <0.01 |
| VitD def. | 4 | 53.9 (34.0, 73.8) | 94.4 | <0.01 | 7 | 51.8 (33.0, 70.5) | 98.1 | <0.01 |

Abbreviations: Ins., insufficiency; def. deficiency; Tetra., tetraplegia; para., paraplegia; C.I., confidence interval.

Supplement Table S8. Subgroup analysis of chronic and acute SCI in meta-analysis

|  | Chronic SCI alone | | | | Involving acute or inpatients | | | |
| --- | --- | --- | --- | --- | --- | --- | --- | --- |
| Symptoms | No. | Estimate (95% C.I.) | I^2^(%) | P | No. | Estimate (95% C.I.) | I^2^(%) | P |
| VitD ins. | 6 | 81.1 (72.5, 89.8) | 93.3 | <0.01 | 6 | 82.0 (73.5, 90.6) | 92.0 | <0.01 |
| VitD def. | 6 | 43.8 (28.5, 59.2) | 96.9 | <0.01 | 4 | 57.7 (35.5, 80.0) | 95.9 | <0.01 |

Abbreviations: Ins., insufficiency; def. deficiency; C.I., confidence interval.
